# Effects of Combining 2 Weeks of Passive Sensory Stimulation with Active Hand Motor Training in Healthy Adults

**DOI:** 10.1371/journal.pone.0084402

**Published:** 2014-01-09

**Authors:** Aija Marie Ladda, Joerg Peter Pfannmoeller, Tobias Kalisch, Sybille Roschka, Thomas Platz, Hubert R. Dinse, Martin Lotze

**Affiliations:** 1 Functional Imaging Unit, Center for Diagnostic Radiology, University of Greifswald, Greifswald, Germany; 2 Neural Plasticity Lab, Institute for Neuroinformatics, Ruhr-University Bochum, Bochum, Germany; 3 BDH-Klinik Greifswald, Neurorehabilitation Centre and Spinal Cord Injury Unit, University of Greifswald, Greifswald, Germany; Emory University, United States of America

## Abstract

The gold standard to acquire motor skills is through intensive training and practicing. Recent studies have demonstrated that behavioral gains can also be acquired by mere exposure to repetitive sensory stimulation to drive the plasticity processes. Single application of repetitive electric stimulation (rES) of the fingers has been shown to improve tactile perception in young adults as well as sensorimotor performance in healthy elderly individuals. The combination of repetitive motor training with a preceding rES has not been reported yet. In addition, the impact of such a training on somatosensory tactile and spatial sensitivity as well as on somatosensory cortical activation remains elusive. Therefore, we tested 15 right-handed participants who underwent repetitive electric stimulation of all finger tips of the left hand for 20 minutes prior to one hour of motor training of the left hand over the period of two weeks. Overall, participants substantially improved the motor performance of the left trained hand by 34%, but also showed a relevant transfer to the untrained right hand by 24%. Baseline ipsilateral activation fMRI-magnitude in BA 1 to sensory index finger stimulation predicted training outcome for somatosensory guided movements: those who showed higher ipsilateral activation were those who did profit less from training. Improvement of spatial tactile discrimination was positively associated with gains in pinch grip velocity. Overall, a combination of priming rES and repetitive motor training is capable to induce motor and somatosensory performance increase and representation changes in BA1 in healthy young subjects.

## Introduction

Training-independent sensory learning protocols have been introduced in order to find alternative approaches to motor training to drive changes on human perception and behavior. The effectiveness of such forms of training-independent sensory learning has been demonstrated in different sensory domains. It has been explained by the fact that the stimulation protocols used are capable to alter synaptic transmission and efficacy [1], [2]. Repetitive electric stimulation (rES) of the fingers is a form of training-independent sensory learning and has been demonstrated to improve tactile perceptual abilities [3], [4] and to drive plasticity processes. Following a single application of rES, individual gains of tactile discrimination were correlated with expansion of blood oxygenation level dependent (BOLD) signals in primary somatosensory cortex (SI) indicating a close link between changes in early sensory areas and overall perceptual performance [5]. rES is similarly effective in elderly individuals thereby resetting the age-related decline of tactile discrimination [6]. Remarkably, rES of the fingers also improved sensorimotor performance in elderly participants [7], [8]. Because of its effectiveness and its ease of use, rES is currently applied in patients after stroke or brain lesion. When applied over weeks or longer, beneficial effects on tactile perception and motor function have been observed [1], [9]. Earlier studies on effects of electric stimulation of the median nerve have demonstrated that this procedure increases hand strength in stroke patients [10]. A combination of tactile stimulation with a one-day session of thumb motor training has been described to enhance training effects in stroke patients [11].

While the effectiveness of repetitive sensory stimulation protocols in adult, aged and brain-injury patients is well-documented, so far no study on the combination of rES with motor training has been published in healthy young adults yet. In addition, there is a controversial discussion about associations between representational map size in primary somatosensory cortex (S1) and primary motor cortex (M1) following shorter or longer periods of motor training. Measurements of BOLD -signal immediately after training demonstrated an increase of BOLD-magnitude in M1 [12]. On the contrary, studies measuring long-term training effects observed a more focused and economized representation map in the primary motor cortex [13], [14]. A similar observation has been made for the visual cortex (V1) following perceptual training: within the first few weeks of visual training, there were increases both in activation in the V1 subregion of the trained visual field quadrant and in task performance. But while performance levels remained high, brain activation in the corresponding areas decreased to baseline levels [15]. We hypothesized that through facilitating effects, even high performing young adults would benefit from a combination of rES and motor training, resulting in a more rapid skill acquisition In addition we wanted to explore the long-term effects on the BOLD-response in relation to tactile discrimination abilities.

## Materials and Methods

To investigate the effect of combining rES with active motor training, 15 strongly right-handed young participants underwent a motor training of their left non-dominant hand. We used the left hand to avoid ceiling effects after training which can be expected when testing the dominant hand [16]. We applied the so-called arm ability training (AAT) because it is repetitive, comprehensive, includes concomitant performance measurement and shows good training effects in stroke patients [17], [18] and healthy subjects [19]. It trains different abilities such as speed, dexterity, aiming and steadiness. Prior to motor training the finger tips of the left hand were electrically stimulated repeatedly over a period of 20 minutes. Motor outcome was tested with three untrained motor dexterity tests and hand grip force measurement for both hands and assessed using the eight trained motor performance tasks from the AAT. Sensory outcome comprised monofilaments and spatial tactile resolution testing for different fingers of both hands. In addition, we tested the BOLD -response to tactile stimulation of the left index finger pre and post training during fMRI to evaluate changes in index finger representation over the training period.

### Participants

We studied 15 right-handed participants each aged 22 to 28 years (age mean = 24.9±2.2 years standard deviation (SD); 7 women). All participants were strongly right-handed (laterality quotient (LQ) = 98.6±3.7; range 89–100) according to the Edinburgh Handedness Inventory [20]. None of the participants suffered from any neurological disorder or vascular disease, nor were they on any regular medication (contraceptives excluded).

Participants were recruited via notice boards at the university campus. Any previous or current regular activity in playing musical instruments was considered exclusion criterion for study participation.

### Ethics Statement

All participants gave their written and informed consent according to the Declaration of Helsinki, and the study was approved by the ethics committee of the Medical Faculty of the University of Greifswald (BB 126/11).

### Experimental schedule

The training period comprised ten consecutive days of training plus one day of rest after the sixth day. On days 1, 2 and 5 training took place in the laboratory in the presence of the instructor. On all the remaining training days participants practiced independently at home. Motor performance was assessed prior to the first training session, on day 5 after the training unit was completed, and one day after the last training session at the end of the second week. Sensory assessment was conducted prior to the first training session and on the day after the last training session as well. fMRI examinations were carried out four days before training started and one day after the last day of training in order to detect long-term effects only and to avoid short term effects of excitability increase following stimulation [21].

### Training

**Figure 1:**
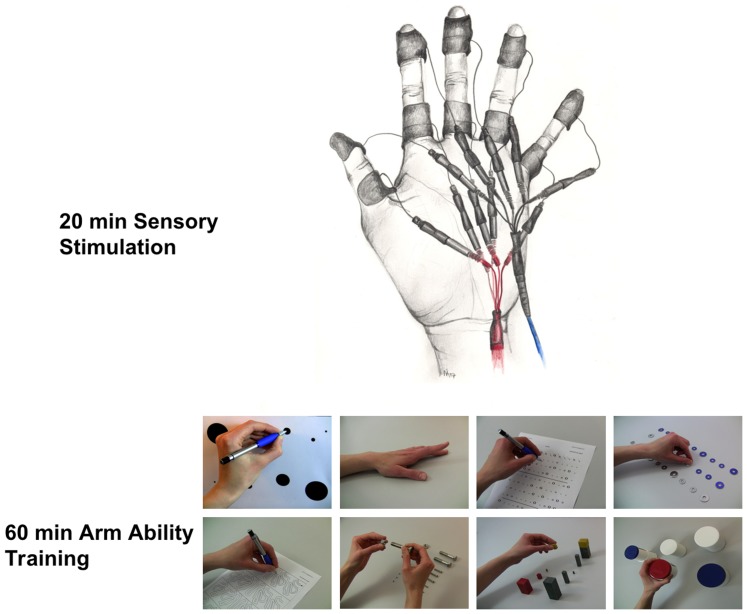
Description of the stimulation and training procedures. Top: Montage of electrodes for repetitive electric stimulation (rES) of the finger tips. Bottom: Eight tasks of the arm ability training (AAT) used for training of the left non-dominant hand: aiming, tapping, crossing, turning coins, labyrinth, bolts and nuts, placing small objects, placing large objects.

We used the Arm-Ability-Training (AAT) [17] for repetitive and comprehensive motor training of the left non-dominant hand. Participants received detailed instructions on the training method and the documentation software that was being used (AFT 1.2; Platz, Greifswald; Programming by OLIOID GmbH, Berlin, Germany). The AAT comprises eight different tasks (see Figure 1), divided in two training sessions overall approximating 60 minutes a day over two weeks (10 days of training). On the first day participants were taught the correct execution of each task while the instructor was responsible for operating the software which documented performance durations of the tasks during training. From the second day on participants had to operate the software autonomously, i.e. pressing the space key to start and stop the integrated stopwatch, while the instructor supervised correct execution of the tasks.

### rES protocol

The participants were stimulated on their left finger tips for 20 minutes/day before they started motor training. The method used was described earlier by Kalisch et al. [7], [8]. The rES sequence consisted of stimulus trains of 1 s (single pulse-duration: 0.2 ms (square), frequency: 20 Hz) and inter-train intervals of 5 s. The sequence was played back from a digital storage device that triggered a standard two-channel TENS device (SM2-AKS, Pierenkemper, Germany) via a custom-made input-channel. The pulses were transmitted via adhesive surface electrodes (1 * 4 cm, Pierenkemper, Germany) fixed on the first and third finger-segment (cathode proximal; see Figure 1). Stimulation intensity was adjusted to the twofold sensory threshold separately for median and ulnar nerve innervated fingers resulting in an average initial stimulation intensity of 10.8±1.5 mA on d1–d3 and 7.8±0.9 mA on d4 and d5.

### Strength and sensory assessment

Maximum grip force [bar] of both hands was assessed using a vigorimeter (Martin Vigorimeter). Three measures were taken and averaged for each time of measurement.

A Grating Orientation Task (GOT) was used as described by Van Boven et al. [22] to assess the tactile acuity threshold for the area of the fingertip. All fingers of the left hand were tested prior to the first training session and approximately 18–24 hours after completion of the last training session in week 2; additional testing of the right middle finger served as a control condition. Nine different types of hemispherical domes were used for assessment, measuring grating distances of between 0.5 and 3.0 mm. For each size type, 16 trials were performed, and testing started with the greatest distance of gratings. Subjects were asked to close their eyes during the test. Gratings were applied to the distant pad of each finger, either horizontally or vertically oriented, resulting in an indentation of approximately 2 mm and lasting for about 1.5 seconds. The participants were required to make an instant statement about the perceived orientation of the gratings. Testing was aborted when the error-rate of 25% was reached.

For assessment of minimal force-detection threshold, Frey-Hair testing was performed for different localizations: on the fingertips of left d1–d5 and on the dorsum of the left hand, in the radial nerve area. The right hand served as a control condition, testing d1 and d3. Participants were asked to close their eyes and report whenever they felt a sensation on their skin. The filaments were pressed against the skin up to three times at a 90° angle until they bowed and were held in place for 1.5 seconds.

### Performance testing of trained tasks

On days 1 (prior to the first training session), 5 and 12 (for 5 and 12 after the training session), performance of each of the eight tasks of the standardized Arm-Ability-Training was assessed measuring the time needed [s] to complete the eight AAT-tasks. The trained and untrained hand were tested in a pseudorandomized order. On the first day of testing each participant was allowed an equal minimum of practice to ensure the execution of the task was understood. The tasks covered four different types of movement: Gross force movements (Placing Heavy Objects), sequential finger movements (Tapping) visually guided (Aiming, Crossing Circles, Labyrinth) and somatosensory guided movements (Turning Coins, Nuts And Bolts, Placing Small Objects). To compare different types of movements with regard to the sensory systems predominantly involved in the execution of the particular task we contrasted averages of primarily visually-guided movements with those of non-visually guided movements (somatosensory-guided movements, sequential finger movements) We hypothesized that after application of rES, tests with predominantly visually guided movements would show less improvement compared to the tests that primarily recruit somatosensory resources.

### Performance testing of not directly trained tasks

To determine fine motor dexterity the Nine-Hole-PEG-Test was performed with right and left hand, measuring the time needed [s] to take nine pegs from a container mold one by one, insert them into nine holes successively, and then remove the pegs and replace them into the mold again.

The Roeder Manipulative Aptitude Test (RMAT) was used to determine speed and dexterity of arm, hand and finger movements (see Roeder Manipulative Aptitude Test Manual, Lafayette Instrument). Test instructions were given in German language. Performance testing of the first (rods and caps) and the second test (washers and nuts) was conducted. The test involving bilateral hand activity was omitted. Each test was practiced in a standardized way, to allow for stable performance. Each hand was tested separately and a pseudorandomized order was used. For statistical calculation, raw scores of the tests were used.

### Statistical analysis

SPSS (V20, IBM Corp., USA) was used for statistical analysis. A 3 time (pre, week 1, week 2) and 2 hand (left, right) repeated measure (RM) ANOVA was conducted to analyze changes of grip force. Somatosensory performance was analyzed by means of 2 time RM-ANOVA, separately for each finger. For trained and untrained motor tasks 3 time (pre, week 1, week 2) and 2 hand (left, right) RM-ANOVA were conducted. The direction of significant effect was tested with t-tests corrected for multiple comparisons [23]. For analysis of the AAT-tasks, averages of similar movement type performance were calculated, resulting in the above mentioned categories of trained movements. We performed correlation analyses of the percentual improvement of different somatosensory-guided motor tasks (Turning Coins, Placing Small Objects, Bolts and Nuts, NHPT, RMAT) and the improvement rates of domes discrimination assessment. Based on a ranking of initial performance-level (averaged for all AAT-tasks) we investigated the influence of initial motor performance on the effect of the training method. Ranking in the individual tasks of the AAT was weighted by a score between 1 and 15, with the highest rank (i.e. the fastest performer) receiving 15 points, permitting an intertask comparison. Adding the scores for individual tasks an overall ranking was conducted for the initial level of AAT-performance. The overall initial performance-level ranking was used as a covariate in the RM-ANOVA evaluating the averaged results of all AAT-tasks. Based on the t-values and considering correlations (r-values) of the data, Cohens' d was used to calculate the effect size.

### MRI Data Acquisition

We used a 3T MRI-scanner (Verio, Siemens, Erlangen, Germany) with a 32 channel head coil. Functional imaging was performed with a standard gradient-echo EPI sequence of 32 transversal slices oriented along the subjects AC-PC plane. In plane resolution was 2×2 mm^2^, slice thickness 3 mm and the gap between slices 1 mm. The field of view was 208×208 mm^2^ corresponding to an acquisition matrix of 104×104. Repetition time was 2 s, echo time 23 ms, and the flip angle 90°. Structural imaging was carried out using a sagittal T1-weighted 3D MPRAGE with 176 slices, a spatial resolution of 1×1×1 mm^3^ and a gap of 0.5 mm between the slices. The field of view was 250×250 mm^2^ corresponding to an acquisition matrix of 256×256. Repetition time was 1690 ms, echo time 2.52 ms, total acquisition time 3∶50 min and the flip angle 9°. In both sequences GRAPPA with a PAT factor of 2 was used.

### Tactile Stimulation and Functional Paradigms during MRI

To investigate the changes in the BOLD-response during tactile stimulation, pneumatic stimulus finger clips (MEG International Services Ltd., Coquitlam, Canada) were used to apply tactile stimuli to the subjects' left index finger tips. The stimulators were composed of a support structure and a membrane, with the membrane measuring about 1 cm in diameter. The stimulators were mounted via the support structure and the membrane was actuated by a computer controlled pneumatic valve. During stimulation, pulses with a length of 50 ms and a variable inter-stimulus interval with an average duration of 300 ms were applied for 10 s, resulting in a stimulation frequency of about 3 Hz, which elicited a feeling of pulsating pressure mainly transmitted by Merkel cells [24]. Followed by a rest period of 10 s, this blocked design was repeated 10 times resulting in a total number of 300 stimulations per finger tip. Identical positioning of the stimulators in the pre- and post-examination was assured by photo documentation of the stimulator position in each session. Stimuli application and scanner synchronization were controlled by Presentation software (Neurobehavioral Systems Inc., Albany, USA). We asked the participants to focus attention on the stimuli presented on the index finger.

### fMRI Data evaluation

Data were analyzed with the FreeSurfer Analysis Software Suite v5.1 [25]. Since only regions in the cortex were of interest, the surface-based stream was used. The structural scans of each subject were reconstructed automatically and separately for the pre- and the post-examination. The functional scans were evaluated using fs-fast. After motion correction the functional images were co-registered to the subject's anatomical scans using boundary based register and are transformed to fsaverage (MNI305). For removing the effect of individual draining vessels [26] we performed a group average over all subjects, which should minimize the effect of vessels due to the variety in their orientation. Since the somatotopy in Brodmann area 3b needs to be evaluated individually [26] and somatotopic differentiation within this area does not stand normalization procedures, data analysis was restricted to Brodmann area 1 (BA1). BA1 processes tactile shape recognition [27] and is included in Freesurfer as an anatomical mask [28]. For restricting the somatotopic region to the index finger [29], a tolerance of one standard deviation was included in the label, to account for the variability in the individual functional representation. The contrast of the activation was computed against baseline using a GLM and the group result is smoothed using a Gaussian filter with a 2 mm isotropic kernel. Results were thresholded using a Bonferroni correction for multiple comparisons over the ROI at a significance level of α = 0.05.

## Results

### Grip force and non-trained tasks

For grip force assessment, repeated measures (RM) ANOVA showed a significant influence for the factor time (F_1,14_ = 5.24, *P*<0.05) but not for the factor hand (F_1,14_ = 2.31, n.s.; no interactions between both factors either: F_1,14_ = 2.92, n.s.). Over both hands, from pre- to post-measurement, grip force increased (two-sided t-test: t_29_ = −3.02, *P*<0.01). Repeated measures ANOVA of the Nine Hole Peg-Test showed significant effects of factors time (F_1,14_ = 11.60; *P*<0.001) and hand (F_1,14_ = 6.00; *P*<0.05) without significant interaction. Both hands showed initially similar performance levels (t_1,14_ = 1.89; n.s.) and both improved over time (left: t_1,14_ = 3.14; *P*<0.01; right: t_1,14_ = 2.52; *P*<0.05). After the intervention performance of the right hand was significantly faster than left-handed performance (t_1,14_ = 3.02; *P*<0.01). For the first task of the Roeder Manipulative Aptitude Test similar effects of factors time (F_1,14_ = 61.22; *P*<0.001;) and hand (F_1,14_ = 79.95; *P*<0.001) were observed, with the right hand at all times performing at a higher level than the left (pre: t_1,14_ = −7.71; *P*<0.001; post: t_1,14_ = −7.09; *P*<0.001) and with both hands increasing performance speed over time (left: t_1,14_ = −5.92; *P*<0.001; right: t_1,14_ = −5.98; *P*<0.001). For the second task of the Roeder Manipulative Aptitude Test, only the factor time was significant (F_1,14_ = 34.46; *P*<0.001), with the right hand displaying better performance only prior to the intervention (t_1,14_ = −2.98; *P*<0.05) and both hands improving over time (left hand: t_1,14_ = −5.60; *P*<0.001; right hand: t_1,14_ = −2.97; *P*<0.001).

### Somatosensory assessment

Tactile acuity increased significantly for left d1 (thumb; RM-ANOVA; d1: F_1,14_ = 7.78, *P*<0.05), whereas the other digits of the left hand or d3 of the right hand showed no significant changes over time (RM-ANOVA; F_1,14_≤0.93, n. s.). As for the effect size Cohen's d was in the middle range for d1 and d3 of the left hand (d1: d = 0.64; d3: d = 0.53). For the remaining digits of the left hand, Cohen's d was low or very low (d2: d = 0.07; d4 = 0.46; d5 = 0.17) and the same was found for d3 of the right hand (d = 0.11).

Performance of the Frey-Hair test revealed no changes over time (RM-ANOVA F_1,14_ = 1.35, n. s.).

### Trained Tasks (AAT-testing)

**Figure 2:**
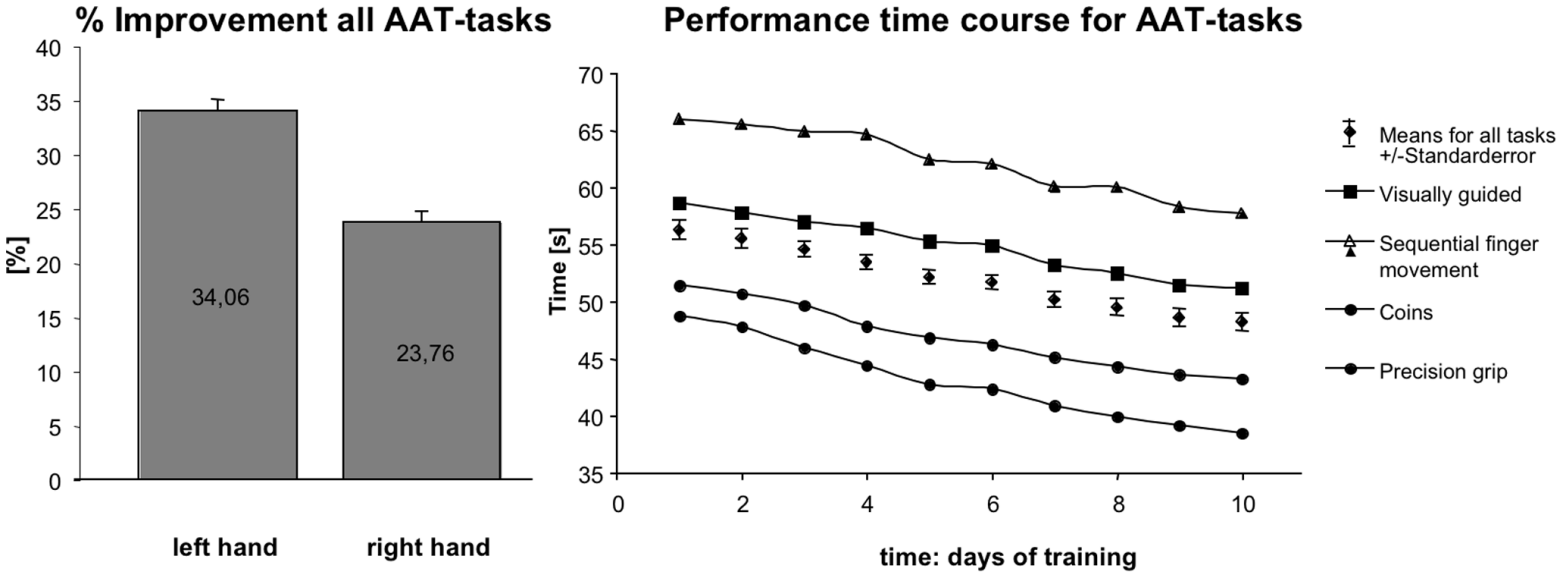
Overview on the performance changes over training time. Left: Average percentual improvement in the AAT-tasks plotted for the trained left and the untrained right hand. Means are provided with standard errors. Right: Detailed absolute increase of performance of the AAT tasks over ten consecutive days for each type of movement trained.

The gain in performance over time for all AAT-tasks and for either hand displayed high improvement rates (left hand: 34.1±1.2%; right hand: 23.8±1.0%; see Figure 2). Repeated measures ANOVA comparing visually-guided, non-visually-guided tasks revealed significant effects of factors time (F_2,28_ = 270.43; *P*<0.001) and hand (F_1,14_ = 67.89; *P*<0.001) on performance time. Significant interactions were found for time*hand (F_2,28_ = 44.98; *P*<0.001), hand*task (F_1,14_ = 42.18; *P*<0.001), and time*task (F_2,28_ = 5.87; *P*<0.01). Subsequently conducted two-sample t-tests revealed significant improvement of performance for all tasks and both hands (visually guided left: t_14_ = 14.88; *P*<0.001; visually guided right: t_14_ = 12.14; *P*<0.001; sensory-guided left: t_14_ = 16.97; *P*<0.001; sensory-guided right: t_14_ = 16.59; *P*<0.001; placing heavy objects left: t_14_ = 10.11; *P*<0.001; placing heavy objects right: t_14_ = 6.09; *P*<0.001). Initial performance levels for the AAT tasks of the right hand were significantly higher than those of the left hand throughout all the tasks (visually-guided: t_14_ = 9.87; *P*<0.001; sensory-guided: t_14_ = 7.82; *P*<0.001; placing heavy objects: t_14_ = 2.78; *P*<0.05). Performance after two weeks of training showed comparable levels for both hands for sensory-guided movements (t_14_ = −1.28; n.s.) and heavy objects (t_14_ = −1.62; n.s.), whereas for visually-guided movements the right hand was still faster than the left hand (t_14_ = 3.47; *P*<0.01).

Cohen's d for these tasks was within significant range for both the left (visually-guided: d = 0.94; sensory-guided: d = 0.95; heavy objects: d = 0.92) and the right hand (visually-guided: d = 0.93; sensory-guided: d = 0.95; heavy objects: d = 0.86).

### Correlation Analysis

**Figure 3:**
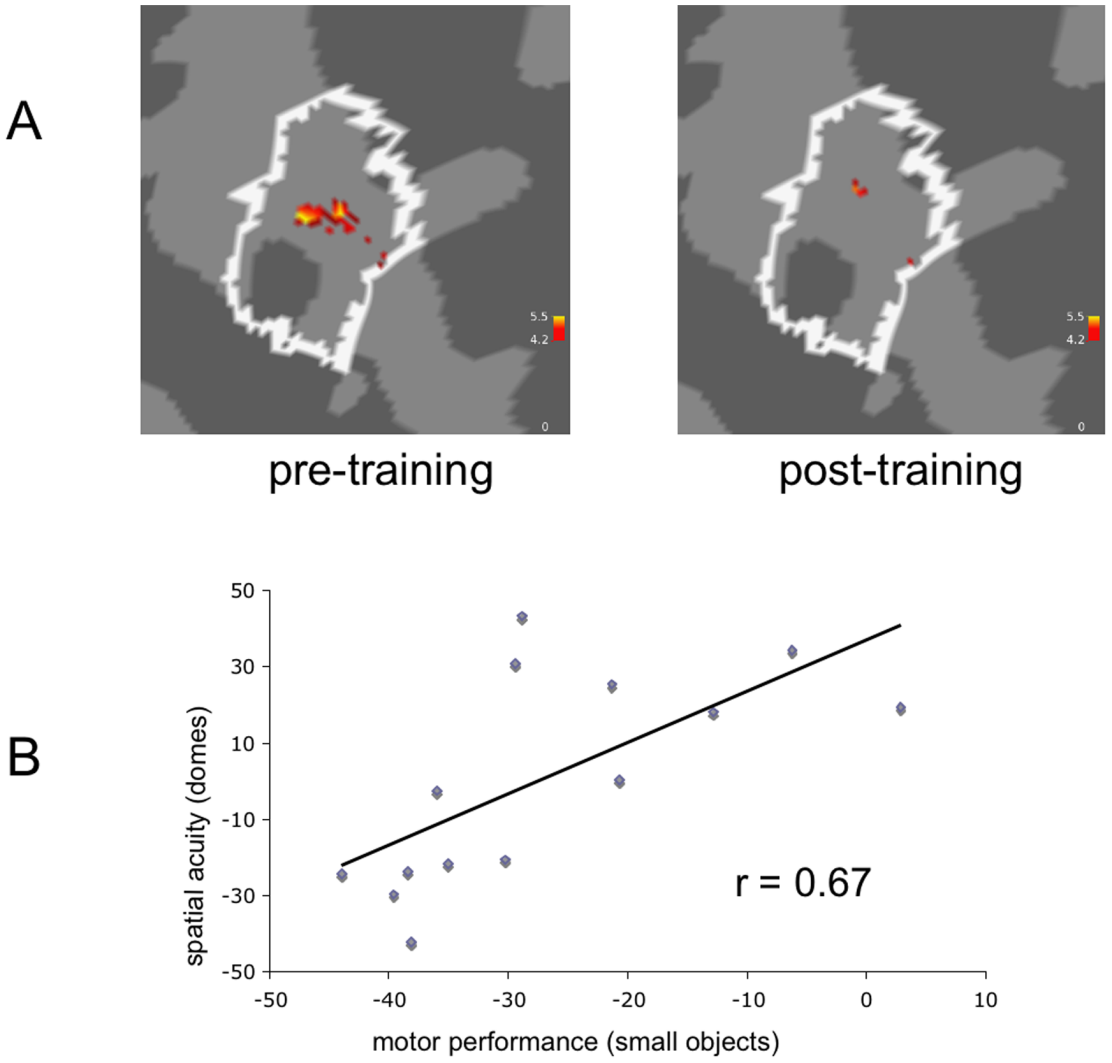
Somatosensory findings on changes over time. A. Visualization of the fMRI-map of the BA 1 activation (ROI indicated with a white line) of the index finger stimulation before (left) and after (right) 2 weeks of intervention (combination of rES and active training). The representational map is decreased in size after two weeks of training although spatial acuity increase was positively associated with increase of pinch grip performance as indicated below. B. The correlation of spatial resolution and motor performance of the AAT task placing small objects was r = 0.67; *P*<0.01.

Increases in discrimination abilities of the left d2 (index finger) as assessed with the domes discrimination task were positively correlated with increases in performance of the AAT-task Placing Small Objects with the left hand (r = 0.67, *P*<0.01; Figure 3). Furthermore lower baseline performance tended to predict a better outcome after training of screwing small objects (r = 0.55; *P*<0.05). A lower activation maximum in BA 1 ipsilateral to the stimulated left index finger prior to training predicted a better training outcome for somatosensory driven movements of the trained left hand (Roeder test part 1: r = 0.53; *P*<0.05).

### Representation size in BA1

During the pre-measurement BOLD-response after repetitive tactile stimulation of the left fingertip showed a large representation area in BA1 of the right hemisphere. After 2 weeks, peak activation decreased only moderately. In contrast, the extent of the representational map (map size) decreased considerably (from about 22 to 3 mm^2^; Figure 3; Table 1) as indicated by a decrease of cluster size by a factor of 7. For the activation maximum we observed a shift in the ROI BA1 of about 3.9 mm which was lower than the effective linear voxel extension of 4 mm and is therefore neglectable.

**Table 1:** Parameters of the clusters found in the contralateral (right hemispheric) BA1 for the pre- and the post-examination.

time	maximal activation [t-value]	size [mm^2^]	MNI-coordinates			cluster-wise significance
			x	y	z	
pre	5.5	21.8	47.9	−24.6	56	3.6
post	4.9	3.1	47.1	−23.7	59.1	1.2

## Discussion

Our study showed that even young adult subjects do profit considerably for left hand motor function after a 2-week training, developed for motor training in stroke patients, with a precedent 20 minute period of electric fingertip stimulation. Interestingly, there was a considerable transfer of performance gain between the trained and untrained hand, which improved right hand performance as well thereby hampering a comparison between trained and untrained hand. As expected, rES in combination with motor training had a positive effect on tactile acuity confirming previous findings [3], [4], [6], [7], [8]. As a result, subtasks requiring cutaneous input (e.g placing small objects, bolts and nuts) benefitted from the combined intervention. At the same time we also observed benefits for both trained and untrained tasks and strength of arm and hand-muscles, in line with the findings of Cohen et al. [10]. Notably, for the trained task “placing heavy objects” the left hand showed more improvement than the right, whereas for the untrained (vigorimeter) task both hands profited equally. At a cortical level, the 2 weeks of training with both rES and AAT did not result in an increase of size of fMRI-representation maps of the index finger as one could predict from single applications, but instead representational maps in BA 1 showed a considerable shrinking in map size.

When applied without prior rES in a former study, the arm ability training (AAT) resulted also in a considerable increase of motor function for the left trained hand in young adult healthy participants [19]. These observations suggest that the AAT might be suited for modeling effects of a comprehensive hand training in healthy young adult participants. The observed transfer to the non-trained right hand was high, in line with studies reporting high transfer of trained motor patterns to the untrained hand [30], [31], which is independent on the hand side trained, i.e. hand dominance but dependent on the mode of transfer [31] and age of the participants [32], underlining the potential for rehabilitation. Most of the tasks investigated have mirror-image properties and are highly susceptible to intermanual transfer.

Small but significant improvements of dexterity using a peg board task have been reported for young adults following a single session of rES without any motor training [33]. Interestingly in our study we found higher improvement in a peg board task (NHPT) for the untrained dominant right hand. To give an explanation for comparable improvement in both hands it would be feasible that the training optimizes recruitment of resources involved in sensorimotor interaction, improving or balancing the communication of the underlying cortical networks. Positive additional effects might be drivable when testing a cohort of less high performing individuals as can be expected in elderly subjects. This hypothesis would also be supported by the association between baseline-performance and percentual performance gain of a task involving high sensorimotor interaction (nuts and bolts). As for the remaining tests, the left hand seemed to profit particularly in the second RMAT task. The time-crucial component of this task consists in picking up small thin washers laying in a mold in a single layer, demanding high-level sensorimotor-interaction, whereas for the first task, fast movements of the fingers make up a major component while picking up the different items makes up the smaller portion of the task.

rES is capable to increase tactile acuity of the finger tips as has been repeatedly demonstrated for young, adult and elderly participants [3], [4], [6], [7], [8]. In the present study, we tested rES in combination with motor training. Therefore, it must remain open, whether the observed increase in acuity of the thumb was due to the additional rES or to the AAT applied alone. During AAT, pinching movements are extensively trained (Figure 1). As a consequence, it is possible that two weeks of AAT affect tactile acuity. Interestingly, pinching movement performance revealed a positive correlation between tactile acuity of the index finger (Figure 3B). A positive association of tactile acuity gain and precise finger pinching movements has been reported after rESin a group of elderly participants [8]. Overall, a combination of AAT with rES might be especially suited for enhancing associations between tactile and motor performance markers, which has to be further investigated in more detail.

In contrast to tactile acuity, touch thresholds as measured with Frey filaments were not altered. This lack of changes of touch threshold has been repeatedly observed in other studies employing rES [33]. It had been argued that the beneficial effects of rES result from changes in synaptic efficacy and synaptic connections. In contrast, touch thresholds seem to reflect predominantly peripheral factors such as mechanoreceptor density and mechanoreceptor composition, which most likely remain unaffected by cortical plasticity processes.

Potential mechanisms of combining rES with active motor training might be related to cortical excitability changes. Cortical excitability is also increased following repetitive transcranial magnetic stimulation (rTMS [34]) or transcranial direct cortical stimulation (TDCS, for a review see [35]). In addition, more focal stimulation strategies through somatosensory electrical nerve stimulation affect cortical excitability [36]. In fact, following rES, SI excitability increases [37], which might enhance effects of a subsequent active motor training.

There is an ongoing debate about cortical economization, habituation effects during repeated measurements and the effect of short- and long-term training on the size of primary representation maps in the somatosensory (S1) and motor (M1) cortex. Very early fMRI studies on repetitive motor training reported a subsequent enlargement of cortical activity in the contralateral M1 during learning (and repetition) of rapid finger movement sequences within the period of a few weeks [38]. Comparably, musicians show an increase of somatosensory representation areas when investigated with magnetoencephalography (MEG) during stimulation of their finger tips in comparison to non-musicians [39]. An enlargement of primary representation areas after training has also been shown specifically for the frequency spectrum of the instrument used in the primary auditory cortex [40]. This long-term training does also result in a more focal representation centered on the contralateral M1 and S1 after years of sensorimotor training [13], [41]. However, there are also short-term changes reported for primary motor cortex, where excitability increases within the first 30 minutes after repetitive motor training [21]. For the same experimental situation increased contralateral M1-representations have been demonstrated using fMRI to record BOLD signals [13]. On the other hand, repeated fMRI-measurements without any training have been reported to show habituation effects with decreased representation map size in the contralateral primary sensorimotor cortex [42]. Overall, our data support findings reported for the motor system in stroke patients [43], that an initially high coactivation of the ipsilateral primary cortex predicts lower performance gain during training and extends it to the somatosensory system.

In our current study we applied elaborate imaging and evaluation techniques using cytoarchitectoral masks for defining representational maps. For the ROIs tested, we observed no relevant change of the highest magnitude of activation. Instead, we found a large change in the extent of the representational area. Both observations are in line with the notion of economization of cortical resources after a long-term combined application of rES and active training. It should be noted that our BOLD analysis was restricted to cortical responses in BA 1 leaving out BA 3b. This was based on Schweitzer and colleagues who found that BA 3b representation shows high differences between subjects and thus a normalized evaluation is not possible. Since after normalization S1-response in BA 3b was absent, our present data confirm the occurrence of high interindividual differences. Instead, normalization was assessed as a necessary procedure for eliminating BOLD from vessels which are a relevant problem in evaluating somatotopic representation in individual brains [44]. In future studies we recommend a further increase of spatial resolution and a combination of MRI-angiography and BOLD-imaging to eliminate BOLD from larger vascular origin from analysis of S1-representation sites. Similarly, further studies are needed to obtain analogous information for a group that underwent active motor training only.

These findings are entirely different to those reported earlier using electric source localization [45] or fMRI and BOLD signal recording [5], [46] to monitor cortical reorganization following single rES application.

A similar dissociation of reorganization pattern has been made for visual cortex (V1) following perceptual training: within the first few weeks of visual training, there were increases both in activation in the V1 subregion of the trained visual field quadrant and in task performance. But while performance is saturated, brain activation in the corresponding areas decreased to baseline levels [15]. These findings across areas and modalities indicate that there might be distinct temporal phases in which the long-term maintenance of perceptual or behavioral alterations is coded in cortical regions beyond the primary areas. This pattern of changes has been captured by the so-called two-stage model [47], [48], according to which plastic changes first develop transiently in early sensory areas, but are then transferred to higher cortical areas, thereby stabilizing the long-term training and learning effects.

### Conclusions

Our results point to an increased training effect of sensory stimulation of the finger tips in advance to repetitive motor training at least for those participants who start with lower pre-training motor performance and in particular for tasks that depend on precise somatosensory feedback. This study however, raises several questions concerning underlying cortical mechanisms especially the interactions within the sensorimotor system and the influence of interhemispherical dysbalances, intermanual transfer, long-term effects and application in elderly patients among others which should be addressed in future studies.
